# Quality of life assessment in facial palsy: validation of the Dutch Facial Clinimetric Evaluation Scale

**DOI:** 10.1007/s00405-015-3508-x

**Published:** 2015-01-28

**Authors:** Ingrid J. Kleiss, Carien H. G. Beurskens, Peep F. M. Stalmeier, Koen J. A. O. Ingels, Henri A. M. Marres

**Affiliations:** 1 Department of Otolaryngology and Head & Neck Surgery, Radboud university medical center, PO Box 9101, 6500 HB Nijmegen, The Netherlands; 2 Department of Orthopedics, Section Physical Therapy, Radboud university medical center, Nijmegen, The Netherlands; 3 Department for Health Evidence, Radboud university medical center, Nijmegen, The Netherlands

**Keywords:** Facial palsy, Assessment, Quality of life, Facial Clinimetric Evaluation Scale, Translation, Validation

## Abstract

This study aimed at validating an existing health-related quality of life questionnaire for patients with facial palsy for implementation in the Dutch language and culture. The Facial Clinimetric Evaluation Scale was translated into the Dutch language using a forward–backward translation method. A pilot test with the translated questionnaire was performed in 10 patients with facial palsy and 10 normal subjects. Finally, cross-cultural adaption was accomplished at our outpatient clinic for facial palsy. Analyses for internal consistency, test–retest reliability, construct validity and responsiveness were performed. Ninety-three patients completed the Dutch Facial Clinimetric Evaluation Scale, the Dutch Facial Disability Index, and the Dutch Short Form (36) Health Survey. Cronbach’s α, representing internal consistency, was 0.800. Test–retest reliability was shown by an intraclass correlation coefficient of 0.737. Correlations with the House–Brackmann score, Sunnybrook score, Facial Disability Index physical function, and social/well-being function were −0.292, 0.570, 0.713, and 0.575, respectively. The SF-36 domains correlate best with the FaCE social function domain, with the strongest correlation between the both social function domains (*r* = 0.576). The FaCE score did statistically significantly increase in 35 patients receiving botulinum toxin type A (*P* = 0.042, Student *t* test). The domains ‘facial comfort’ and ‘social function’ improved statistically significantly as well (*P* = 0.022 and *P* = 0.046, respectively, Student *t*-test). The Dutch Facial Clinimetric Evaluation Scale shows good psychometric values and can be implemented in the management of Dutch-speaking patients with facial palsy in the Netherlands. Translation of the instrument into other languages may lead to widespread use, making evaluation and comparison possible among different providers.

## Introduction

Patients experiencing peripheral facial palsy experience both functional and psychosocial consequences. The evaluation of both aspects is fundamental in the management of facial palsy. Among the consequences of peripheral facial palsy are brow ptosis, incomplete eye closure (leading to exposure keratopathy), external nasal valve collaps, oral incompetence, speech and articulation problems, synkinesis (involuntary movement during voluntary movement), esthetic impairments, and the inability to express emotions, sometimes leading to social isolation.

Assessment of facial function in peripheral facial palsy comprises different perspectives; evaluation by a physician using grading scales [1, 2], objective (sometimes automated) measurement methods [3–5], and patient self-assessment using questionnaires. In an era of rapid developments in computerized, automated measurement tools, the influence of the disease on the patient’s quality of life must not be overlooked, and should be considered an essential feature of clinical assessment, and remains important during first consultation, during follow-up, and after treatment.

The self-assessment of patients using questionnaires gives an impression of the influence of disease on quality of life. For this purpose, nondisease-specific questionnaires exist [6, 7], as well as disease-specific questionnaires, though very few of them are adapted in regular clinical practice. Kahn et al. [8] developed an instrument which covers both the functional and psychosocial aspect of facial palsy, the Facial Clinimetric Evaluation Scale (FaCE Scale). This questionnaire consists of 15 questions with a 5-point Likert scale. The FaCE Scale comprises six domains; facial movement, facial comfort, oral function, eye comfort, lacrimal control, and social function. Total and domain scores range from 0 (worst) to 100 (best).

The FaCE Scale is a valid, reliable, and easily administered instrument [8]. Since its original description, this questionnaire has been used in several studies showing patient satisfaction following treatment [9–12]. We wanted to implement this instrument in the Dutch-speaking population in the Netherlands, both because we want to use an instrument that covers both functional and psychosocial domains and also so that we may compare our treatment and recovery results with international results.

In the current literature there is no consensus on ‘gold standard’ guidelines for translating quality of life questionnaires. Two methods are described: the forward–backward translation [13–15] and the dual panel translation [16]. Dual panel translation compromises the translation by a team of translators working together and assessment of the translation by a lay panel [17]. The forward–backward translation seems to be the most accepted method, although there is no evidence to support this view. Acquadro et al. [17] performed a literature review in 2008; they did not find evidence in favor of one method, but strongly advised researchers to adopt a multistep approach. When using a questionnaire in another country and another language, translation of the items alone is not enough. The items must be adapted to the new culture to maintain the content validity of the instrument: cross-cultural adaption is required. [18, 19].

The aim of this study was to create a Dutch version of the FaCE Scale and to test its internal consistency, test–retest reliability, construct validity, and responsiveness for a valid use in the Dutch language and culture.

## Materials and methods

### Translation

The study protocol was assessed according to guidelines of the local committee on research involving human subjects; no formal ethical review was required.

**Fig. 1:**
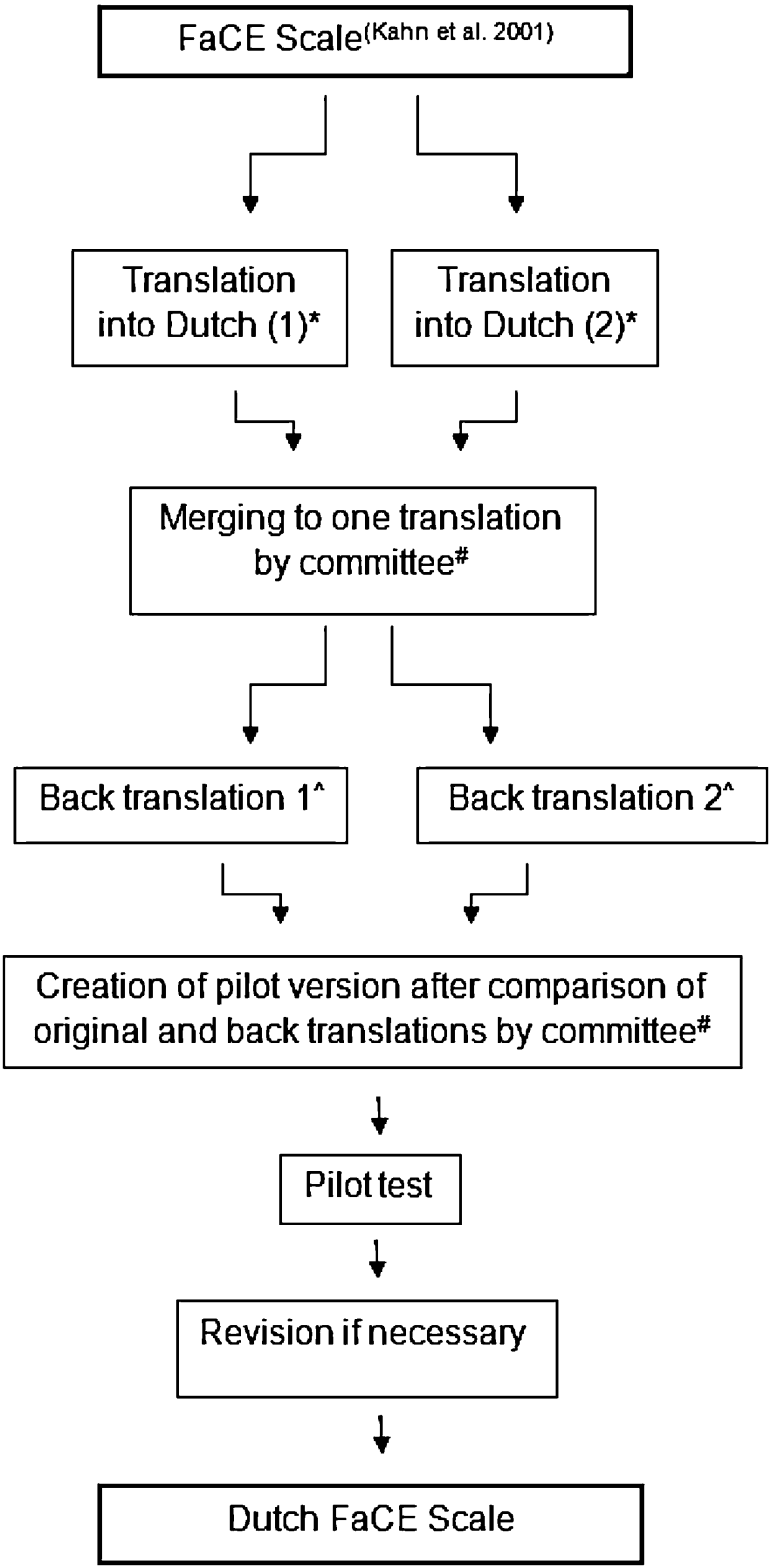
Method of translation in this study: ^*^ Two independent translators, both native Dutch with American–English as their second fluent language; one of them was a medical doctor. ^#^ Committee consisting of the authors of this manuscript. ^^^ Two independent translators: both of American origin with Dutch as a second language and blinded for the original questionnaire; one of them had a medical background

We approached the developers of the FaCE Scale and obtained permission to use the instrument for translation and validation [8]. A forward–backward translation approach was used in this study (Fig. 1). Considerations and difficulties of each step were documented. Choice of wording and phraseology had to be compatible with a reading level of age 14 [13]. The pilot test was performed in a group of ten patients with a facial palsy and ten persons without history of facial disease. Respondents completed the questionnaire and were asked about difficulties with answering and understanding the items. After this pilot test, final adjustments were made and documented.

### Validation

Validation of the Dutch FaCE Scale was performed at our university medical center between December 2012 and August 2014. Dutch-speaking adult (18 years or older) patients with a facial palsy were included. Patients completed three different questionnaires: (1) the Dutch FaCE Scale, and to test construct validity (2) the Dutch Facial Disability Index (FDI), and (3) the Dutch Short Form (36) Health Survey (SF-36). All responses were entered in IBM SPSS Statistics 20 (IBM Corp. Armonk, NY) according to the principle of double data entry. In addition, gender, age, etiology, side and duration of the palsy, House-Brackmann (HB) scores, and Sunnybrook (SB) scores were collected in the database. This information was retrieved from the medical charts retrospectively, so missing data could occur. Patients not receiving any form of treatment were sent the Dutch FaCE Scale again after 2 weeks for test–retest reliability. At the end of the study, to increase the response rate for test–retest, patients were sent the Dutch FaCE Scale (plus FDI and SF-36) 2 weeks before visiting our clinic and the test–retest was performed during their visit, independent in the waiting room. Patients receiving treatment with botulinum toxin type A for synkinesis were sent the Dutch FaCE Scale 4 weeks after injection to test the responsiveness of the questionnaire.

### Facial Disability Index

The FDI is a disease-specific quality of life questionnaire for patients with facial palsy, developed at the Facial Nerve Center in Pittsburg around 1996 by VanSwearingen et al. [20]. The FDI has two domains: physical function and social/well-being function. The physical function scores range from −25 (worst) to 100 (best), and the social/well-being function scores range from 0 (worst) to 100 (best). This questionnaire has been translated into Dutch according to a forward–backward method previously (not published), but has not officially been validated for use in the Dutch culture.

### The short form (36) health survey

The SF-36 is a general health-related quality of life questionnaire, consisting of 36 questions. All questions save one (item 2) are converted in eight domains: physical functioning (PF), role limitations due to physical health problems (RP), bodily pain (BP), general health perceptions (GH), vitality (VT), social functioning (SF), role limitations due to emotional problems (RE), and mental health (MH). The scores range from 0 (worst) to 100 (best). This self-report health status questionnaire is the most widely used instrument and has been translated for use in more than 40 countries (including Dutch) [6, 21].

### Statistical analysis

IBM SPSS Statistics 20 (IBM Corp. Armonk, NY) was used for data collection and statistical analysis. First, descriptive analyses were performed to show patient characteristics. Cronbach’s *α* coefficient was calculated to test the internal consistency of the Dutch FaCE Scale. Intra-class correlation was calculated to analyze test–retest reliability. Correlations between the Dutch FaCE Scale and the HB score, SB score, FDI, and SF-36 were calculated using Spearman’s rank correlation coefficient to show construct validity. To test responsiveness, a paired samples *t*-test was performed. [22].

## Results

### Pilot testing

Ten normal subjects, without history of facial disease, completed the pilot version of the Dutch FaCE Scale; they all had a FaCE score of 100 (best score). Ten patients with peripheral facial palsy completed the pilot version of the translated questionnaire as well. Subjects did not document any difficulties in understanding or answering the items, and no further changes were made in the Dutch FaCE Scale.

### Validation

Between December 2012 and September 2014, 93 patients completed the Dutch FaCE Scale, FDI, and SF-36. Patient characteristics are shown in Table 1.

**Table 1:** Patient characteristics

	*n*	%	Mean	SD	Median	Range
Gender						
Female	61	66				
Male	32	34				
Age (years)			55.1	13.8	55	20–89
Side						
Left	43	46				
Right	47	51				
Bilateral	3	3				
Time since onset (months)			45	52	29	4–298
Etiology						
Bell’s Palsy	48	52				
Ramsay Hunt	16	17				
Iatrogenic	7	8				
Acoustic neuroma	6	7				
other	16	16				
House-Brackmann			3.3	1.2	3.0	1–6
Sunnybrook			45.9	20.4	49.0	0–83

The category “other etiologies” comprised Lyme disease, congenital facial palsy, traumatic cases, parotid malignancies, cholesteatoma, and benign facial nerve tumors. FaCE scores are shown in Table 2.

**Table 2:** FaCE Scores (total and domains)

	Mean	SD	Median	Range	*n*
Total Score	44.6	16.4	46.7	3–87	83
Facial Movement Score	33.0	22.3	33.3	0–83	81
Facial Comfort Score	32.2	25.6	25	0–100	83
Oral Function Score	45.6	27.1	50	0–100	83
Eye Comfort Score	41.8	33.6	43.8	0–100	82
Lacrimal Control Score	47.4	28.7	50	0–100	78
Social Function Score	62.3	26.1	62.5	0–100	83

The internal consistency of the Dutch FaCE Scale was tested by Cronbach’s *α*, which showed a value of 0.80. A Cronbach’s *α* >0.7 is generally considered acceptable, and *α* > 0.8 as good [22]. The Cronbach’s *α* scores ranged from 0.57 to 0.84 for the FaCE Scale sub domains (Table 3). Test–retest reliability was calculated with the intra-class correlation coefficient (ICC). Forty patients met the criteria for test–retest reliability analysis, but only 21 patients (53 %) completed the questionnaires at both time points. Results are shown in Table 3. Test–retest reliability was demonstrated with ICC’s ranging from 0.65 to 0.80.

**Table 3:** Internal consistency reliability and test–retest reliability

	Internal consistency		Test–retest	
	Cronbach’s *α*		ICC	95 % CI
	Test	Retest		
Total Score	0.80	0.81	0.737	0.463–0.883
Facial Movement Score	0.64	0.54	0.653	0.322–0.843
Facial Comfort Score	0.84	0.77	0.802	0.564–0.917
Oral Function Score	0.79	0.90	0.700	0.341–0.872
Eye Comfort Score	0.57	0.43	0.747	0.472–0.891
Lacrimal Control Score	#	#	0.741	0.427–0.895
Social Function Score	0.75	0.85	0.674	0.350–0.854

*ICC* intraclass correlation coefficient, *CI* confidence interval, # this sub domain has only one item, for internal consistency *n* = 93, for test–retest reliabilty *n* = 21

Correlations between the FaCE scores and the FDI, SF-36, HB, and SB scores are shown in Tables 4 and 5. Correlation with the HB score is negative because of the design of the HB (1 is no palsy, 6 is complete flaccid palsy). A Spearman’s correlation coefficient of 0.570 for the SB score indicates good construct validity of the Dutch FaCE Scale. As expected, the HB and SB scores show the best correlations with the facial movement domain of the Dutch FaCE Scale (*r* = −0.410 and *r* = 0.603, respectively). The total FaCE score correlates well with the FDI physical function and FDI social/well-being function scores; *r* = 0.713 and *r* = 0.575, respectively. The FDI social/well-being function has the highest correlation with the FaCE social function domain (*r* = 0.729). The FDI physical function has the highest correlation with the FaCE oral function domain (*r* = 0.661). The SF-36 domains correlate best with the FaCE social function domain, with the strongest correlation between the both social function domains (*r* = 0.576). FaCE domain facial movement shows the weakest correlations with the SF-36. Since the SF-36 is a general health-related questionnaire, strong correlations were not expected.

**Table 4:** Correlation between FaCE scores with House-Brackmann scores, Sunnybrook scores, and Facial Disability Index (Spearman’s correlation coefficient)

FaCE scores	House-Brackmann (*n* = 62)	Sunnybrook (*n* = 54)	FDI physical function (*n* = 92)	FDI social/well-being function (*n* = 92)
Total	−0.292*	0.570**	0.713**	0.575**
Facial movement	−0.410**	0.603**	0.310**	0.062
Facial comfort	0.134	0.086	0.443**	0.318**
Oral function	−0.222	0.385**	0.661**	0.365**
Eye comfort	−0.226	0.475**	0.367**	0.108
Lacrimal control	0.006	0.128	0.247*	0.180
Social function	−0.119	0.323*	0.477**	0.729**

**Table 5:** Correlation between FaCE scores with SF-36 (Spearman’s correlation coefficient)

FaCE scores	Physical function	Physical role	Bodily pain	General health	Vitality	Social function	Emotional health	Mental health
Total	0.394**	0.389**	0.270**	0.276**	0.447**	0.555**	0.382**	0.416**
Facial movement	0.163	−0.046	−0.062	−0.110	−0.010	0.227*	0.140	0.072
Facial comfort	0.153	0.269*	0.291**	0.164	0.302**	0.283**	0.248*	0.310**
Oral function	0.280**	0.310*	0.084	0.118	0.341**	0.336*	0.254*	0.207*
Eye comfort	0.115	0.138	0.136	0.121	0.062	0.127	0.005	0.055
Lacrimal control	0.141	0.169	0.167	0.157	0.180	0.134	0.045	0.133
Social function	0.391**	0.376**	0.274**	0.346**	0.519**	0.576**	0.432**	0.499**

** *P* < 0.01, * *P* < 0.05, *n* = 86

### Responsiveness

Thirty-five patients received treatment for synkinesis with botulinum toxin A. Nineteen of them (54 %) had received botulinum toxin previously, and the other 46 % were new to this treatment. Total FaCE score before treatment was 44.7 (SD 15.0) and about 4 weeks after treatment 48.5 (SD 15.2). This difference is statistically significant (*P* = 0.042, Student *t*-test). The domains ‘facial comfort’ and ‘social function’ improved statistically significantly as well (*P* = 0.022 and *P* = 0.046, respectively, Student *t*-test).

## Discussion

In this study, the FaCE Scale has been translated and validated for use in the Netherlands. Good psychometric values for the Dutch version of the self-assessment questionnaire are shown. The internal consistency of the Dutch FaCE Scale is reflected by a Cronbach’s *α* of 0.80. The internal consistency of the Swedish and German translations shows a Cronbach’s *α* of 0.92 and 0.91, respectively [23, 24]. A possible explanation for this difference might be a different patient population used in the different studies. We compared our patient characteristics with the Swedish and German study, and they match highly. Other methodological differences between studies can explain different outcomes as well, for example, if questionnaires were completed individually or in company of a physician. We consider our internal consistency as good, as well as the test–retest reliability, and construct validity.

### Strength of this study

Translation of the FaCE Scale into the Dutch language and validation for use in the Dutch culture were performed according to the highest standards for translation of self-assessment questionnaires [14, 19, 25].

### Limitations of this study

Translators (forward and backward) were neither professional translators, nor experienced in questionnaire translation, and not familiar with the questionnaire. During both stages, a translator with a medical background and a layperson were chosen; the idea was to produce one translation that would reflect the concepts of the original questionnaire and the other translation would reflect the language used by a layperson. In the literature there is no consensus on the choice of translators [17].

The FDI we used in this study has not been translated and validated for use in Dutch according to the current standards. We could have done this together with the translation and validation of the FaCE Scale; however, we have chosen to validate just one questionnaire. Assessment of the health-related quality of life by the use of two self-assessment questionnaires seems unnecessary. The FaCE Scale is the instrument of our choice, based on the study of Kahn et al. and Ho et al. [8, 26]. Kahn et al. [8] showed that the mean difference in FDI social/well-being function scores between subjects with facial palsy and control subjects was relatively small, indicating that the FDI instrument does not discriminate as well as the FaCE Scale between normal and disease states. Ho et al. performed a systematic review of patient-reported outcome measures in facial palsy. Three self-assessment questionnaires met their inclusion and exclusion criteria: the FaCE Scale, the FDI, and a questionnaire developed by Borodic et al. [9]. The FaCE Scale met the most psychometric standards [26].

One of the domains of the Dutch FaCE Score (eye comfort) shows a poor internal consistency (*α* = 0.57); in the validation study of Kahn et al., this domain has the lowest score as well (0.72). A possible explanation for our low α might be the sample in which the questionnaire was applied; reliability is a characteristic of the test scores, not of the test itself; our group might be more heterogeneous in terms of co morbidity, for example [27].

The response rate for test–retest reliability was quite low in this study; 40 patients met the criteria for test–retest reliability analysis, which meant they did not receive any form of treatment during their visit and received a second Dutch FaCE Scale per mail after 2 weeks. Only 21 of them (53 %) completed the questionnaires. We likely could have increased this response rate if we had been more persistent in pursuing a response.

### Comparison with grading systems

Many different facial grading systems have been developed, almost all focusing on the physiological and anatomical abnormalities in the face [28, 29]. The effect of the disability on the patient’s quality of life is not covered by these systems, while reduced social functioning after facial palsy is described in the literature [30]. The extent of physiological impairment is not by definition correlated with impact on patient quality of life. For example, a patient with a HB 5 can have very little influence of the palsy on his/her quality of life, whereas a patient with HB 2 can be completely socially isolated. Kahn et al. [8] found that the correlations between the FaCE Scale and physician-graded scales were not always as expected; for example, the eye comfort domain of the FaCE Scale did not strongly correlate with the physician’s assessment regarding eye closure, suggesting that the degree of eye closure does not predict the problems the patient experiences.

## Conclusion

The Dutch FaCE Scale is a valid, reliable, and easy-to-perform instrument for the assessment of the influence of facial palsy on the patient’s quality of life. The use of the Dutch FaCE Scale can now be implemented in the management of patients with facial palsy in our clinic. With comparable studies in Sweden, China, and Germany, this self-assessment questionnaire for patients with facial palsy is now available in five languages [8, 23, 24, 31]. This is a great step forward in the implementation of a widely used instrument.
